# Second Language Word Learning through Repetition and Imitation: Functional Networks as a Function of Learning Phase and Language Distance

**DOI:** 10.3389/fnhum.2017.00463

**Published:** 2017-09-28

**Authors:** Ladan Ghazi-Saidi, Ana Ines Ansaldo

**Affiliations:** ^1^Department of Communication Disorders, University of Nebraska at Kearney Kearney, NE, United States; ^2^School of Speech Therapy and Audiology, University of Montreal Montreal, QC, Canada; ^3^Centre de Recherche de l’Institut Universitaire de Gériatrie de Montréal Montreal, QC, Canada

**Keywords:** verbal repetition, functional connectivity, networks, language and cognitive control, L2 word learning, task complexity, novelty, salience

## Abstract

**Introduction and Aim**: Repetition and imitation are among the oldest second language (L2) teaching approaches and are frequently used in the context of L2 learning and language therapy, despite some heavy criticism. Current neuroimaging techniques allow the neural mechanisms underlying repetition and imitation to be examined. This fMRI study examines the influence of verbal repetition and imitation on network configuration. Integration changes within and between the cognitive control and language networks were studied, in a pair of linguistically close languages (Spanish and French), and compared to our previous work on a distant language pair (Ghazi-Saidi et al., 2013).

**Methods**: Twelve healthy native Spanish-speaking (L1) adults, and 12 healthy native Persian-speaking adults learned 130 new French (L2) words, through a computerized audiovisual repetition and imitation program. The program presented colored photos of objects. Participants were instructed to look at each photo and pronounce its name as closely as possible to the native template (imitate). Repetition was encouraged as many times as necessary to learn the object’s name; phonological cues were provided if necessary. Participants practiced for 15 min, over 30 days, and were tested while naming the same items during fMRI scanning, at week 1 (shallow learning phase) and week 4 (consolidation phase) of training. To compare this set of data with our previous work on Persian speakers, a similar data analysis plan including accuracy rates (AR), response times (RT), and functional integration values for the language and cognitive control network at each measure point was included, with further L1-L2 direct comparisons across the two populations.

**Results and Discussion**: The evidence shows that learning L2 words through repetition induces neuroplasticity at the network level. Specifically, L2 word learners showed increased network integration after 3 weeks of training, with both close and distant language pairs. Moreover, higher network integration was observed in the learners with the close language pair, suggesting that repetition effects on network configuration vary as a function of task complexity.

## Introduction

Verbal repetition refers to articulating a word after hearing it (Moritz-Gasser and Duffau, 2013). This process is important in language acquisition, both developmentally and in learning a second language (L2), and has applications in language rehabilitation.

Repetition and imitation is one of the oldest methods used to teach an L2. Since 1631, repetition has been seen as the most effective way to learn an L2 for functional use; ever since then, the most successful methodologies in L2 teaching and learning have included repetition and imitation of words and sentences to a greater or lesser extent (Celce-Murcia, 2001). However, repetition and drilling have also been heavily criticized, particularly after the introduction of cognitive approaches, when drilling by repetition was considered mechanical and meaningless (Haycraft, 1978; Richards and Nunan, 1990; Cross, 1995; Larsen-Freeman and Anderson, 2013).

The use of repetition as a learning tool stems from behaviorism (Watson, 2013; Skinner, 2014), which argues that the environment affects human behavior, and advances the idea that learning occurs as a result of repeated exposure to a given stimulus. This view has been criticized for attributing a passive role to the learner, while ignoring active internal factors in the learning process (Watson, 2013). Behaviorists have claimed that many scholars misunderstand behaviorism and its concepts (Skinner, 1974).

In recent years, repetition has received a lot of attention and several studies have focused on its role in different types of learning (e.g., Bygate et al., 2013; Horst, 2013). Repetition has been used and proven to be beneficial in language treatment for children with developmental language disorders and adults suffering from aphasia after stroke or head trauma (Kempler and Goral, 2011). In particular, repetition, imitation and drilling are popular among L2 teachers and learners, at least for word learning and accurate pronunciation, since they are helpful and can support language learners in achieving functional communication in daily life (Berthier and Lambon Ralph, 2014).

The use of repetition to enhance neuroplasticity has been vindicated by recent studies on the recovery of function following brain damage (Crosson et al., 2007). Among the methodological approaches used to examine the links between repetition and functional neuroplasticity, Hope et al. (2014) used a multifactorial approach and identified both linguistic and non-linguistic processing areas involved in word repetition. They identified which linguistic and non-linguistic processing areas are involved in word repetition and categorized them in eight different processing groups. The authors showed that areas underlying word repetition include both language processing areas, similar to the model proposed by Price (2012), and general processing areas. Further, the results of many studies of healthy participants and clinical rehabilitation studies addressing the effect of repetition on the learning of novel stimuli suggest that related neuroplastic changes are associated with cognitive control (e.g., Carey et al., 2005; Kimberley et al., 2010; Berthier and Lambon Ralph, 2014).

In addition, it has been demonstrated that complexity is a factor that can impact repetition-induced neuroplasticity, particularly neuroplasticity relevant to cognitive control components (Sadato et al., 1996; Wulf and Shea, 2002; Hlustík et al., 2004; Carey et al., 2005). Evidence for the complexity of repetition processes comes from anatomical and functional neuroimaging studies and computational modeling and shows that repetition is sustained by auditory-motor integration processes, with a supporting hub located at the Sylvian fissure at the parietal–temporal boundary (SPT). Damage to the SPT results in poor performance on word repetition, even in the absence of white matter damage (Hickok et al., 2003; Rogalsky et al., 2015). Together, the evidence shows that repetition entails complex integrated processes, involving language, executive and motor cognitive and neural systems.

Among the neuroimaging tools used to study repetition processes, tractography has been quite popular and has proven informative. In their study, Duffau et al. (2005) showed the importance of the dorsal pathway involving the arcuate fasciculus and the superior longitudinal fasciculus in allowing the conversion of auditory input stored in working memory into phonological-articulatory representations sustaining repetition. This evidence is in line with Hickok and Poeppel’s (2007) model, which included a bilateral ventral stream involved in speech comprehension, and a left-hemisphere-dominant dorsal stream that maps acoustic speech signals to frontal lobe articulatory networks. Along the same lines, Saur et al. (2008) described a dorsal tract involved in mapping of auditory input to motor plans during repetition, and Berthier et al. (2012) showed the importance of the ventral pathway and the arcuate fasciculus (Berthier and Lambon Ralph, 2014) in repetition. Moreover, in their study, Moritz-Gasser and Duffau (2013) strongly supported an interaction between the ventral and dorsal pathways; they claimed that, while auditory stimuli are stored in working memory and sent to the articulation areas through the dorsal pathway, the ventral pathway contributes to semantic processing.

The role of the dorsal pathway in repetition was already hypothesized by Wernicke (1874). Lichtheim (1885) translated Wernicke’s idea into a diagram. The anatomical correlates of these perspectives (the arcuate fasciculus) were identified years later by Monakow, and accepted by Wernicke in 1908 (Catani and Mesulam, 2008; Glasser and Rilling, 2008). Recent tractography studies have revealed that the temporal and frontal areas are connected through a direct Broca-Wernicke and an indirect Broca-inferior parietal lobule-Wernicke pathway (Catani and Mesulam, 2008; Glasser and Rilling, 2008).

While white matter tractography is extremely informative for understanding the neural correlates of language tasks such as repetition, the functional dimension of these connections remains to be understood. For example, it is well accepted that the brain regions sustaining performance on a given task are not necessarily linked by fiber tracts. Thus, brain regions may be anatomically segregated but nevertheless activated in the context of a given task. Moreover, while activation maps provide information on the average level of involvement of different brain regions involved in the task, information about how these regions constitute networks whose configuration may vary depending on the task is another matter, to be addressed by functional connectivity methodologies (Rogers et al., 2007). Network functional connectivity methods allow one to describe how a given set of relevant areas may cooperate to perform a given task at a given moment in time. Moreover, changes in network configurations as a function of different factors can be addressed by looking at network integration. Thus, changes in integration levels within and between networks cooperating on a given task are estimations of the ease of communication or information flow among these networks and their respective components (Rubinov and Sporns, 2010).

Several studies have looked at between-network integration to explain language processing. For example, Makuuchi and Friederici (2013) used dynamic causal models to examine levels of integration between core language and general domain (working memory) networks for syntactic processing, and reported increased levels of integration between the two target networks with increased processing load.

With regard to word repetition, functional connectivity studies are limited. However a few studies have looked at changes to structural and functional connectivity in the process of word learning. Specifically, functional connectivity and DTI studies suggest that learning new words requires fast and efficient interaction between the frontal and the temporal lobes (López-Barroso et al., 2013). Also, both functional (Yang et al., 2015) and structural differences in connectivity across individuals have been related to variability in word learning success (Catani et al., 2007). With regards to functional connectivity studies, the evidence suggests that four dorsal and ventral networks including motor, frontal, temporal and parietal areas are associated with word learning, with variable degree of engagement through learning phases (López-Barroso et al., 2015). In particular, the dorsal auditory-premotor network has been reported to show more strength and association with individual performance immediately after word learning (López-Barroso et al., 2015). Finally, functional connectivity studies on word learning in an informal learning context, and comparing learners and non-learners, report stronger connectivity between the left and right supramarginal gyri in learners than for non-learners (Veroude et al., 2010). Also, learners seem to rely more on language network, which is better integrated with other networks to process tonal and lexical information of target L2 words (Yang et al., 2015).

The relationship between frontal and temporal cortical areas has been measured by regional cerebral metabolic rates of glucose using positron emission tomography (PET) in word repetition (Karbe et al., 1998). Significant correlations connected frontal and temporal areas with the left planum temporale as a hub, bilaterally.

PET scanning was used in another functional connectivity study to compare network interactions for word and non-word repetition in illiterate participants and compare network interactions for non-words between illiterate and literate participants (Petersson et al., 2000). Differences between the two comparisons included interaction differences in the general domain processing areas and control network. Literate subjects also showed differences in the attentional network and connections between Broca’s area and the inferior parietal region. Thus, word and non-word repetition engages both language and control networks and the level of engagement of the two networks differ in literate and illiterate participants. These results may suggest that the level of engagement of the control network depends on how complex the task is for each group, based on the amount of previous repetitive exposure to similar stimuli (i.e., literate people have more repetitive exposure to words and non-words than illiterate people).

Exposure to similar stimuli, among other factors, depends on L1-L2 linguistic distance. Unlike linguistically distant languages, languages that belong to the same linguistic family and have the same root (e.g., French and Spanish) share many structural similarities (Aitchison, 1999; Finch, 2005). Cross-linguistic similarity, refers to what the learner perceives to be similar (Kellerman, 1977; Odlin, 1989; Ringbom, 2007), and is an important modulating factor in L2 learning (Ringbom, 2007) by affecting the magnitude of learning (Corder, 1979). Since learners “already have a potential vocabulary” (Ringbom, 2007, p.11), L2 words that are similar to L1 equivalents do not require much effort for being stored in the mental lexicon and may be easily activated in L2 production (Ringbom, 2007). Neuroimaging evidence suggests that learning L1-L2 similar words require less cognitive resources including less cognitive control (Ghazi-Saidi and Ansaldo, 2016).

The results of functional connectivity studies suggest that verbal repetition is sustained by both language processing and general domain cognitive control abilities (Berthier et al., 2017). In our previous work (Ghazi-Saidi et al., 2013), we examined network integration changes within and between the language and cognitive control networks in a group of Persian native speakers (L1) learning French words (linguistically distant L2). This work showed that network integration levels within and between language and cognitive control networks decreased with increased L2 proficiency.

The present study goes a step further in this regard, by looking at the regulatory effects of language distance as a complexity factor that modulates repetition effects in network configuration. To do so, by comparing the results of the present study and the previous study (Ghazi-Saidi et al., 2013), and adding further analyses in both groups, we examined changes in integration level between the cognitive control and language networks as a function of language distance between L1 and L2. Language distance is one of the least investigated aspects of L2 neuroplasticity effects (Ghazi-Saidi et al., 2012; Li et al., 2014).

## Materials and Methods

### Experimental Design

In a longitudinal functional magnetic resonance imaging (fMRI) group study, 24 learners of French (L2) were tested for network configuration after short-term (a week) and long-term (4 weeks) audiovisual repetition training to learn L2 words, which consisted in practicing repetition and imitation of new French words (*n* = 130). Behavioral measures and functional connectivity integration values were computed at each measure point and compared across measure points and languages.

### Participants

The participants were 12 (6 females and 6 males) healthy native Spanish-speaking (L1) adults, aged between 26 and 66 (*M* = 40.2, SD = 12.1), and 12 (6 females and 6 males), healthy native Persian-speaking (L1) adults, aged between 26 and 66 (*M* = 40, SD = 21.2) who were assessed for cognitive and learning ability as measured by the Montreal Cognitive Assessment (MoCA; Nasreddine et al., 2005), the Memory and Learning Test (Grober and Buschke, 1987; Grober et al., 1988), and the Stroop test (Beauchemin et al., 1996). Participants showed no significant differences within or between groups on their Stroop scores, reflecting equal cognitive control skills. Participants had no history of neurological or psychiatric disorders. Both groups included equal numbers of men and women (6 each, 12 in total) and were matched for age, education and elementary L2 level. All participants were recruited from the first-level immersion courses offered by the Quebec government for immigrants motivated to learn French, after a rigorous placement test. Participants consented after clear descriptions of the study procedure. Participants responded to a thorough questionnaire based on the work of Paradis and Libben (1987), Flege (1999) and Silverberg and Samuel (2004), which had been used in our previous studies (Scherer et al., 2012; Ghazi-Saidi et al., 2013, 2015; Ghazi-Saidi and Ansaldo, 2016). All participants matched for the amount of their exposure to French, mother tongue and English during the 4 weeks of training; they all attended the same full time French course, listened to the same amount of television/radio and held the same amount of interaction with speakers of French, L1 and English. None of the participants was fluent in English but given that at least some exposure to English is inevitable, participants’ proficiency in English was controlled (i.e., low proficiency), and stimuli were controlled for similarity to English. None of the participants had any knowledge of a fourth language.

### Training and Procedure

Participants started a self-training audiovisual course using a CD containing a homemade computerized word-learning program through verbal repetition and imitation. This program had been used in our previous studies (Ghazi-Saidi et al., 2013, 2015; Ghazi-Saidi and Ansaldo, 2016). It was adapted to each group by controlling for potential cross-linguistic transfer effects between L1 and L2 stimuli.

Stimuli were 130 color images of objects and their names in French pronounced by a French native speaker. Similarity to L1s was controlled across languages (French-Persian and French-Spanish) by including equal numbers of cognates (semantically and phonological similar words cross-linguistically), clangs (phonologically similar words cross-linguistically), and non-cognates (semantically similar words cross-linguistically), and also controlling for similarity to English to avoid cross-linguistic transfer effects (Ringbom, 2007).

Word frequency and length (number of phonemes and syllables) were matched across languages (French, Spanish and Persian). Images were matched for visual complexity, word and object familiarity in all three languages. The category effect was controlled for by selecting an equal number of items in each semantic category including household objects, animals, fruits and vegetables, stationery, and clothing and accessories (Caramazza and Shelton, 1998).

The procedure was as follows: in detailed explanations, participants were instructed to look at the image, listen to the corresponding word, and repeat and imitate the target word, with the purpose of learning to name the picture, as similarly to the model as possible. All participants practiced for 15 min a day, for a total of 28 consecutive days. Participants were frequently contacted by email and telephone to make sure that participants completed all sessions of practice and followed the instructions.

### Data Acquisition

At each measure point, participants performed an overt naming task of all trained items during fMRI scanning. They were asked to lie on their back, with their head immobilized by cushions and belts. Oral answers were recorded by an fMRI-compatible microphone placed close to their mouth. Bite-bars were not used, given the evidence on their effect on attention and performance (Heim et al., 2006). Online movement correction was used for rigid-body head movements. Participants viewed the stimuli via a mirror. Using Presentation software v.11.2^1^, stimuli were displayed on a large monitor reflected in the mirror. Each picture was presented for 4000 ms, followed by a blank page presented for a randomized interval of 4600–8600 ms, followed by the next image. As in our previous studies (Ghazi-Saidi et al., 2013, 2015; Ghazi-Saidi and Ansaldo, 2016), a variable inter-stimulus interval was used to assure better sampling of the hemodynamic response and prevent attentional bias (Huettel et al., 2004). Responses were recorded by Sound Forge software (Sonic Foundry, Madison, WI, USA). Participants were instructed to name the images as fast and accurately as they could. The total duration of the task was 47 min, including 21 min to perform the task in each language. Anatomical acquisition took 5 min.

Acquisition parameters were the same as in previous studies by our team; TR = 3 s, TE = 40 ms, matrix = 64 × 64 voxels, FOV = 24 cm, and slice thickness = 5 mm (Raboyeau et al., 2010; Ghazi-Saidi et al., 2013, 2015; Ghazi-Saidi and Ansaldo, 2016). Sequential slices were used, to avoid the stripping that might happen due to some types of head motion (Siemens 3T Scanner User Training: Supporting Information and FAQ). Acquisition included 28 slides in the axial plan, so as to scan the whole brain, including the cerebellum. A high-resolution structural scan was obtained between the two functional runs (L1 naming task and L2 naming task), using a 3D T1-weighted pulse sequence (TR = 13 ms, TE = 4.92 ms, flip angle = 25°, 76 slices, matrix = 256 × 256 mm, voxel size = 1 × 1 × 1 mm, FOV = 28 cm).

Imaging data were recorded in the Unité Neuroimagerie Fonctionnelle at the Institut Universitaire de Gériatrie de Montréal. The ethics committee of Réseau de Neuroimagerie du Québec has approved this study.

### Data Analysis

All responses were rated by a French native speaker for naming and accent accuracy. Only correct responses were included in the analysis. At both measure points, with both L1 and L2, behavioral measures including accuracy rates (AR) and response times (RT) of oral responses, were calculated using SPSS, version 17.0. A paired-samples *t*-test was conducted to compare the AR (AR^T2^ − AR^T1^) and the RT (RT^T2^ − RT^T1^), at the two points in time (T1: after a week; T2: after 4 weeks).

The data were preprocessed with SPM5^2^ software. SPM5 is the only version that is compatible with NetBrainWork, the software used to calculate functional connectivity. Preprocessing included correcting images for delay in slice acquisition and rigid-body head movements; they were then realigned and smoothed. For each subject, outputs of the SPM5 realignment function were checked for translation (parallel to the *x*-, *y*- and *z*-axes), and rotation around these axes (pitch, roll and yaw), to discard the data from participants with more than 4 mm of head motion (Ghazi-Saidi et al., 2013, 2015; Ghazi-Saidi and Ansaldo, 2016).

Functional connectivity analysis focused on two target networks: the language and cognitive control networks, as described by Price (2010, 2012) and Abutalebi and Green (2007, 2012, 2016), respectively. In the present study, functional integration values within and across these networks were calculated with NetBrainWork software^3^ (Perlbarg et al., 2009). Specifically, for the language network, 21 regions of interest (ROI) were selected based on the model proposed by Price (2010), following a thorough meta-analysis of neuroimaging studies on language processing. Given that the present paradigm used an oral naming task, only those areas reported to be significantly activated with single-word tasks were included in the target language network, namely the middle temporal gyrus, bilaterally, the left posterior superior temporal gyrus, the bilateral temporal pole, the left angular gyrus, the left inferior frontal gyrus, the left middle-frontal gyrus, the pars opercularis (BA 44), the pars triangularis (BA 45), the inferior frontal sulcus, the left ventral pars opercularis, the left dorsal pars opercularis, the left rolandic operculum, the left pars orbitalis (BA 47); the pre-supplementary motor area, the precentral gyrus, the insula, the left putamen, and the hippocampus, bilaterally (Price, 2010). ROIs in the cognitive control network were selected with reference to Abutalebi and Green (2007, 2012, 2016). Thus, there were 11 ROIs in this network, namely the left fusiform gyrus, the left and right postcentral gyri, the right superior parietal lobule, the left and right cingulate gyri, the left anterior cingulate, the left and right inferior frontal gyri, the right limbic lobe, the parahippocampal gyrus, the left frontal lobe and the superior frontal gyrus.

Functional connectivity was calculated by measuring network integration, a value extracted from BOLD data, and represented as a t-map, for all functional networks, reproducible across all subjects. ROIs were identified in the MNI standard space and included in the calculation of integration values; for each ROI, a statistical map with the highest t-score was selected. Then, the extension of the corresponding ROI was achieved by using a region-growing algorithm that recursively added to the region the adjacent voxel with the highest t-score. The algorithm stopped when the region size was 10 voxels (Ghazi-Saidi et al., 2013).

CORSICA (Perlbarg et al., 2007) was used to remove physiological noise from the fMRI data, after which the two networks of interest (NOI) were extracted from the averaged fMRI time-series for the 32 ROIs (21 ROIS in the language network and 11 ROIs in the cognitive control network, as described above). Then functional connectivity was calculated between the NOIs (i.e., the language and control networks). Functional connectivity is the temporal correlation between ROIs. The total correlation, which summarizes the correlation coefficients of all ROIs (in this study, 32) in a single number, is called the integration value (Coynel et al., 2009). The integration values between and within networks were calculated (Marrelec et al., 2008).

A hierarchical model in a Bayesian framework with a numerical sampling scheme was used to account for intra- and inter-subject variability (Marrelec et al., 2006). The samples were then used to provide approximations of probabilities (e.g., probability of an increase in integration between the shallow and consolidation phases, based on the frequency of integration increase observed in the sample). Inferences regarding differences in integration were conducted at a probability of difference higher than 0.90 (Ghazi-Saidi et al., 2013). A fixed-effects group approach was used to infer probable integration values from the data and a Bayesian group analysis with numerical sampling scheme (1000 samples per estimate for these analyses; Marrelec et al., 2008).

During the sampling procedure, the covariance matrix for each group (the group of subjects at either level of proficiency) was estimated, resulting in 1000 estimates of each measure (total integration, between integration and within integration) for each group.

**Figure 1:**
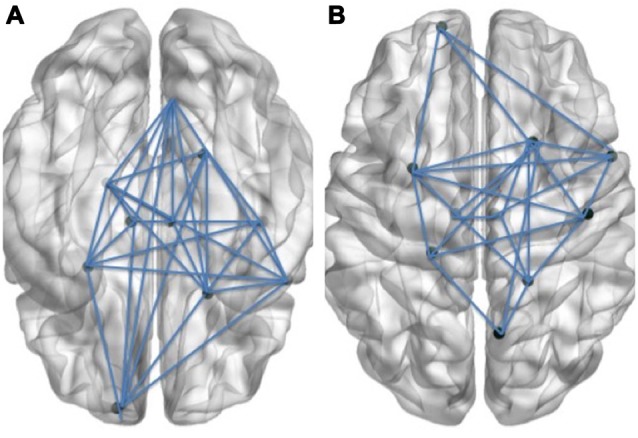
Schematic visual illustration of the Language **(A)** and Cognitive control **(B)** networks. In these schematic figures, the right hemisphere of the brain is shown on the right side of each figure and the left hemisphere is shown on the left side.

Means and standard deviations (SD), or probabilities of an increase between low and high levels of proficiency, were approximated, and an approximation of the frequency of that increase observed in the sample was calculated. This procedure had previously been used by Coynel et al. (2009), Schrouff et al. (2011) and Boly et al. (2012). Thus, the means and SDs for integration reported here correspond to the means and SDs of the integration sample distributions. The probability of an assertion such as [integration_phase 2 > integration_phase 1] is given between 0 and 1, and is considered significant if higher than 0.9. In cases where the probability is lower than 0.1, the complementary assertion ([integration_phase 2 < integration_phase 1]) is true (Ghazi-Saidi et al., 2013). For simplicity’s sake, an equation is used to symbolize the total integration (I) of the network involved in L2 naming: I_Total_ = I_Intra_L_ + I_Intra_C_ + I_Inter_L-C_. In this equation, I_Intra_L_ stands for integration within the language network areas, I_Intra_C_ stands for integration within the control network areas, and I_Inter_ for integration between the two networks (Ghazi-Saidi et al., 2013; Figure 1). The above procedure was performed for each language separately. The total integration value, the integration within language network areas, and the integration within control network areas, as well as the integration between the two networks, were compared between the L1 and L2 in each group, namely the Persian speakers and the Spanish speakers. Thus, the comparisons included Spanish native speakers/Phase 1: L2 vs. L1; Persian native speakers/Phase 1: L2 vs. L1; Spanish native speakers/across phases: L2 and L1. The results will be discussed in the context of the results of our previous study on Persian native speakers/across phases: L2 and L1 (Ghazi-Saidi et al., 2013).

## Results

### Behavioral Results

Both groups showed improved performance in L2 naming after 4 weeks of repetition of L2 words. Persian native speakers were faster than Spanish speakers at phase 2, but this difference did not reach significance (*p* = 0.06). More specifically, at phase 2, the Spanish native speaker group named L2 words significantly faster (*M* = 2.00, SD = 0.50) and more accurately (*M* = 90.17%, SD = 8.2) than in phase 1 (*M*_RT_ = 2.23, SD_RT_ = 0.53), (*M*_AR_ = 69.87%, SD_AR_ = 30.99). The paired-sample *t*-test shows that there was a significant difference between the two phases of evaluation (Phase 2 – Phase 1) both for RTs, *t*_(12)_ = 2.69, *p* = 0.01, and for ARs, *t*_(12)_ = –4.78, *p* = 0.000. As the control condition, the same analysis was computed for L1. Although there was no significant difference, *t*_(11)_ = –1.78, *p* = 0.1, between AR of naming in phase 1 (*M* = 97.05%, SD = 3.4) and phase 2 (*M* = 98.5, SD = 1.61), words were named significantly faster, *t*_(11)_ = 3.45, *p* = 0.006, in phase 2 (*M* = 2.01, SD = 0.59) than in phase 1 (*M* = 2.21, SD = 0.61).

**Figure 2:**
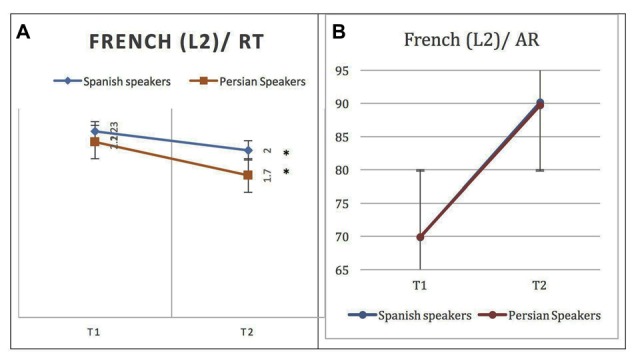
Behavioral results for naming L2 (French) words at phase 1 (after a week) and phase 2 (after 4 weeks) in native Persian and Spanish speakers: **(A)** Response time (RT) in seconds (s). **(B)** Accuracy rate (AR) in percentage. A significant difference is observed across phases for RT but AR overlaps. Error bars show one standard deviation (SD) from the mean. Asterisks (*) indicate that the result is statistically significant.

Similarly, in the Persian native speaker group, at phase 2 L2 words were named significantly faster (*M* = 1.7, SD = 0.23) and more accurately (*M* = 89.74%, SD = 5.3%) than in phase 1 (*M* = 2.1, SD = 0.32), (*M* = 69.9%, SD = 22.85%). The paired-sample *t*-test showed that there was a significant difference between the two phases, for both RTs, *t*_(12)_ = 4.52, *p* = 0.001, and ARs, *t*_(12)_ = –3.02, *p* = 0.012. See Figure 2 for behavioral results.

L1 words were named faster in phase 2 (*M* = 2.21, SD = 0.61) but the difference in RT was not statistically significant, *t*_(12)_ = 3.45, *p* = 0.006. Also, there was no significant difference between phase 1 and phase 2 ARs, *t*_(12)_ = –1.77, *p* = 0.107.

### Functional Connectivity Results

The total integration (Marrelec et al., 2008) value was calculated by adding partial integration values of the language and cognitive control networks, namely I_Total_ = I_Intra_L_ + I_Intra_C_ + I_Inter_ where I = integration, I_Intra_L_ = Intra-integration of language areas, I_Intra_C_ = Intra-integration of cognitive control areas, and I_Inter_ = integration between the two networks (language processing and cognitive control).

#### Phase 1

At phase 1, both the Spanish and Persian groups showed significant differences in integration values across languages, in both the within- and between-language and cognitive control comparisons.

Specifically, the integration value between the language and control networks was lower in L1 than in L2 for both groups. See Table 1 for a summary of results for each group.

**Table 1:** Probability value of the network (significant) differences between L1 and L2 (French) in the Spanish-speaking group and the Persian-speaking group.

L1 vs. L2 (French)	I_Total_	I_Inter_L_	I_Inter_C_	I_Intra_
L1: Spanish	P _L2 > L1_ = 0.998	P _L2 > L1_ = 0.919	P _L2 > L1_ = 0.974	P _L2 > L1_ = 1
L1: Persian	P _L2 > L1_ = 1	P _L2 > L1_ = 1	P _L2 > L1_ = 0.915	P _L2 > L1_ = 1

*P = probability value, I = integration, I_Intra_L_ = Intra-integration of language areas, I_Intra_C_ = Intra-integration of cognitive control areas and I_Inter_ = integration between the two networks (language processing and cognitive control). A probability greater than 0.9 is considered significant*.

#### Spanish Native Speakers: L2 vs. L1

In the Spanish native group, the comparison of networks across languages (L1 and L2) at the low level of proficiency (phase 1) yielded the following results. The total integration value for the language network and the control network in Spanish (L1) was measured as I_Total_ (*M* = 4.312, SD = 0.077) and for French (L2) as I_Total_ (*M* = 4.697, SD = 0.114), and the probability of differences was L2 > L1 = 0.998.

The total within-system integration value for the language network and the control network, for Spanish as L1 was measured as I_Intra_total_ (*M* = 3.280, SD = 0.066) and for the L2, French, as I_Intra_total_ (*M* = 3.484, SD = 0.096); the probability of differences was L2 > L1 = 0.961. The within-system integration value for the language network for Spanish (L1) was I_Intra_L_ (*M* = 3.096, SD = 0.063) and for French (L2) it was (*M* = 3.247, SD = 0.092); the probability of differences was L2 > L1 = 0.919. The value for the within-system integration the control network for Spanish (L1) was measured as I_Intra_C_ (*M* = 0.183, SD = 0.014) and at the high level of proficiency (phase 2) as (*M* = 0.237, SD = 0.023); the probability of differences was L2 > L1 = 0.974.

The total between-systems integration value for the language network and the control network for Spanish (L1) was measured as I_Inter_ (*M* = 1.033, SD = 0.032) and for French (L2) as (*M* = 1.213, SD = 0.047); the probability of differences was L2 > L1 = 1.000.

**Figure 3:**
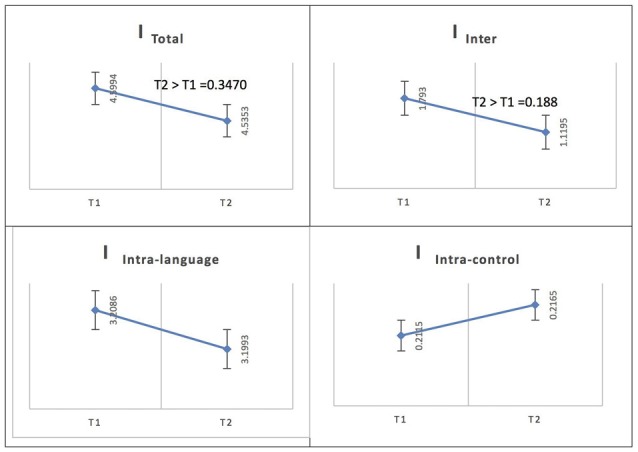
Probability value of the network integration differences between L1 and L2 (French) in the Spanish-speaking group. I = integration, I_Total_ = total integration value, I_Intra_L_ = Intra-integration of language areas, I_Intra_C_ = Intra-integration of cognitive control areas and I_Inter_ = integration between the two networks (language processing and cognitive control). A probability greater than 0.9 is considered significant.

See Figure 3 for an illustration of network integration in phase 1 in Spanish speakers, and across L1 (Spanish) and L2 (French).

#### Persian Native Speakers/Phase 1: L2 vs. L1

In the Persian native group, the comparison of networks across languages (L1 and L2) at the low level of proficiency (phase 1) yielded the following results. The total integration value for the language network and the control network in Persian (L1) was measured as I_Total_ (*M* = 4.0318, SD = 0.0762) and for French (L2) as I_Total_ (*M* = 4.8104, SD = 0.1165) and the probability of differences was L2 > L1 = 1.000.

For Persian (L1), the total within-system integration value for the language network and the control network was measured as I_Intra_total_ (*M* = 3.119, SD = 0.0659) and for French (L2) as I_Intra_total_ (*M* = 3.601, SD = 0.099); the probability of differences was L2 > L1 = 1.000. The within-system integration value for the language network for Persian (L1) was I_Intra_L_ (*M* = 2.925, SD = 0.063) and for French (L2) it was (*M* = 3.369, SD = 0.094); the probability of differences was L2 > L1 = 1.000. The value for the within-system integration of the control network for Persian (L1) was measured as I_Intra_C_ (*M* = 0.193, SD = 0.014) and at the high level of proficiency (phase 2) as (*M* = 0.231, SD = 0.023); the probability of differences was L2 > L1 = 0.915.

The total between-systems integration value for the language network and the control network for Persian (L1) was measured as I_Inter_ (*M* = 0.913, SD = 0.028) and for French (L2) as (*M* = 1.209, SD = 0.043); the probability of differences was L2 > L1 = 1.000.

**Figure 4:**
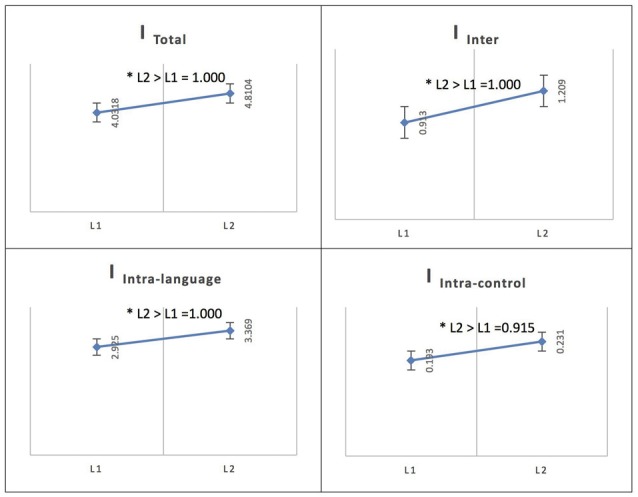
Probability value of the network integration differences between L1 and L2 (French) in the Persian-speaking group. I = integration, I_Total_ = total integration value, I_Intra_L_ = Intra-integration of language areas, I_Intra_C_ = Intra-integration of cognitive control areas and I_Inter_ = integration between the two networks (language processing and cognitive control). A probability greater than 0.9 is considered significant. Asterisks (*) indicate that the result is statistically significant.

See Figure 4 for an illustration of L2 vs. L1 network integration in phase 1 in Persian speakers.

#### Across Phases

Spanish native speakers showed no integration changes over time for any of the studied network comparisons, in either language. See Table 2 for a summary of results across phases.

**Table 2:** Probability value of the network (significant) differences across phases for L1 and L2 (French) in the Spanish-speaking group (L1 = Spanish).

T1 vs. T2	I_Total_	I_Inter_L_	I_Inter_C_	I_Intra_
L2: French	P _T2 > T1_ = 0.998	P _T2 > T1_ = 0.919	P _T2 > T1_ = 0.974	P _T2 > T1_ = 1
L1: Spanish	P _T2 > T1_ = 1	P _T2 > T1_ = 1	P _T2 > T1_ = 0.915	P _T2 > T1_ = 1

*T1 = phase 1 (after a week), T2 = phase 2 (after 4 weeks), P = probability value, I = integration, I_Intra_L_ = Intra-integration of language areas, I_Intra_C_ = Intra-integration of cognitive control areas and I_Inter_ = integration between the two networks (language processing and cognitive control). A probability greater than 0.9 is considered significant*.

#### Spanish Native Speakers/Across Phases: L2

In the Spanish native group, for French (L2), the total integration value for the language network and the control network at the low level of proficiency (phase 1) was measured as I_Total_ (*M* = 4.7096, SD = 0.1132) and at the high level of proficiency (phase 2) as I_Total_ (*M* = 4.7901, SD = 0.1198); the probability of differences was phase 2 > phase 1 = 0.6970. At the low level of proficiency (phase 1), the total within-system integration value for the language network and the control network was measured as I_Intra_total_ (*M* = 3.494, SD = 0.096242) and at the high level of proficiency (phase 2) as I_Intra_total_ (*M* = 3.6028, SD = 0.10265); the probability of differences was phase 2 > phase 1 = 0.7840. The within-system integration value for the language network at the low level of proficiency (phase 1) was I_Intra_L_ (*M* = 3.2560, SD = 0.0920) and at the high level of proficiency (phase 2) it was (*M* = 3.3762, SD = 0.0986); the probability of differences was phase 2 > phase 1 = 0.8220. The value for the within-system integration of the control network at the low level of proficiency (phase 1) was measured as I_Intra_C_ (*M* = 0.2380, SD = 0.0231) and at the high level of proficiency (phase 2) as (*M* = 0.2266, SD = 0.0210); the probability of differences was phase 2 > phase 1 = 0.3630. The total between-systems integration value for the language network and the control network at phase 1 was measured as I_Inter_ (*M* = 1.2156, SD = 0.0465) and at phase 2 as (*M* = 1.1873, SD = 0.0465); the probability of differences was phase 2 > phase 1 = 0.339.

**Figure 5:**
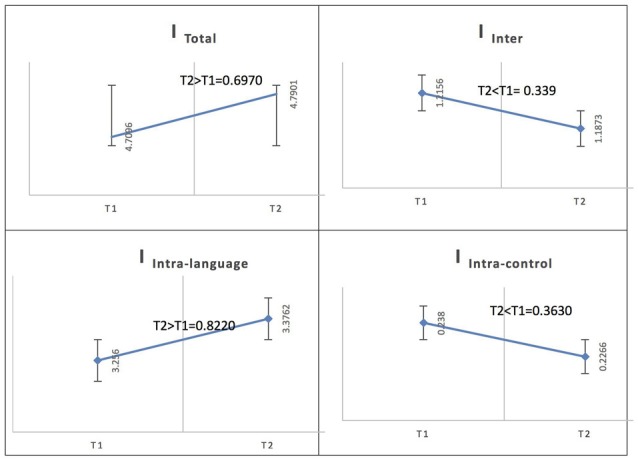
Probability value of the network integration differences across phases for L2 (French) in the Spanish-speaking group (L1 = Spanish). I = integration, I_Total_ = total integration value, I_Intra_Language_ = Intra-integration of language areas, I_Intra_Control_ = Intra-integration of cognitive control areas and I_Inter_ = integration between the two networks (language processing and cognitive control). A probability greater than 0.9 is considered significant.

See Figure 5 for an illustration of network integration across phases for L2 in Spanish speakers.

#### Spanish Native Speakers/Across Phases: L1

For Spanish (L1), the total integration value for the language network and the control network at the low level of proficiency (phase 1) was calculated as I_Total_ (*M* = 4.5994, SD = 0.1127) and at the high level of proficiency (phase 2) as I_Total_ (*M* = 4.5353, SD = 0.1159); the probability of differences was phase 2 > phase 1 = 0.3470. The total within-system integration value for the language network and the control network at the low level of proficiency (phase 1) was measured as I_Intra_total_ (*M* = 3.4201, SD = 0.096245) and at the high level of proficiency (phase 2) as I_Intra_total_ (*M* = 3.4158, SD = 0.099456); the probability of differences was phase 2 > phase 1 = 0.4820. The within-system integration value for the language network at phase 1 was I_Intra_L_ (*M* = 3.2086, SD = 0.0919) and at phase 2 it was (*M* = 3.1993, SD = 0.0953); the probability of differences was phase 2 > phase 1 = 0.4590. The value for the within-system integration the control network at phase 1 was measured as I_Intra___C_ (*M* = 0.2115, SD = 0.0212) and at phase 2 as (*M* = 0.2165, SD = 0.0212); the probability of differences was phase 2 > phase 1 = 0.5590. The total between-systems integration value for the language network and the control network at the low level of proficiency (phase 1) was measured as I_Inter_ (*M* = 1.793, SD = 0.0453) and at the high level of proficiency (phase 2) as (*M* = 1.1195, SD = 0.0451); the probability of differences was phase 2 > phase 1 = 0.188.

According to the Bayesian statistics, to infer that A > B, P (A > B) > threshold = 0.9 and to infer that A < B, P (B > A) = 1 – P (A > B), 1 – threshold = 0.1. Thus, according to the results, for French (L2), as in Spanish (L1), the total integration value (I_Total_), the total within-system integration value for the language network and the control network (I_Intra_total_), the within-system integration value for the language network (I_Intra_L_), the value for the within-system integration of the control network (I_Intra___C_) and the total between-systems integration value for the language network and the control network (I_Inter_) did not change significantly as the level of proficiency increased (phase 1 vs. phase 2).

**Figure 6:**
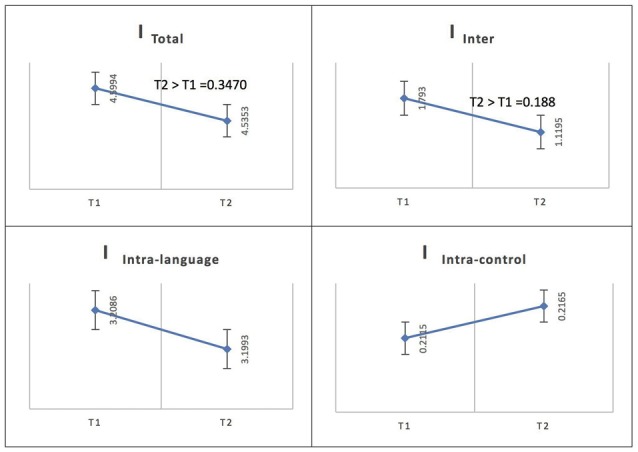
Probability value of the network integration differences across phases for L1 (Spanish) in the Spanish-speaking group. I = integration, I_Total_ = total integration value, I_Intra_Language_ = Intra-integration of language areas, I_Intra_Control_ = Intra-integration of cognitive control areas and I_Inter_ = integration between the two networks (language processing and cognitive control). A probability greater than 0.9 is considered significant.

See Figure 6 for an illustration of network integration across phases for L1 in Spanish speakers.

#### Persian Native Speakers/Across Phases: L1 and L2

The Persian native speaker group showed significantly increased integration values for total, between- and within-network integration levels across phases in L2, whereas the integration value remained unchanged across phases in L1; for details, see Ghazi-Saidi et al. (2013).

**Figure 7:**
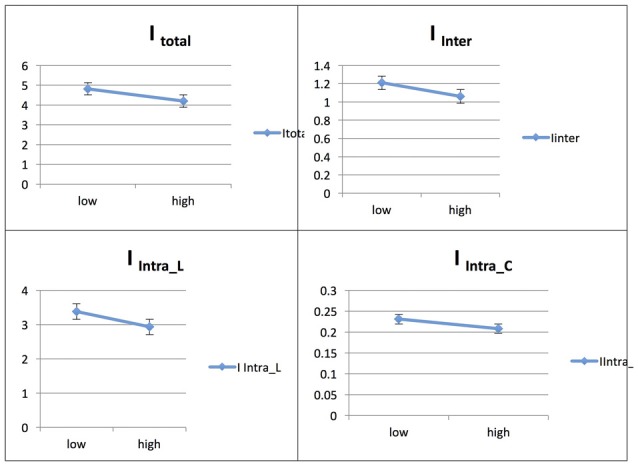
Probability value of the network integration differences across phases for L2 (French) in the Persian-speaking group (L1 = Spanish). I = integration, I_Total_ = total integration value, I_Intra_Language_ = Intra-integration of language areas, I_Intra_Control_ = Intra-integration of cognitive control areas and I_Inter_ = integration between the two networks (language processing and cognitive control). A probability greater than 0.9 is considered significant.

**Figure 8:**
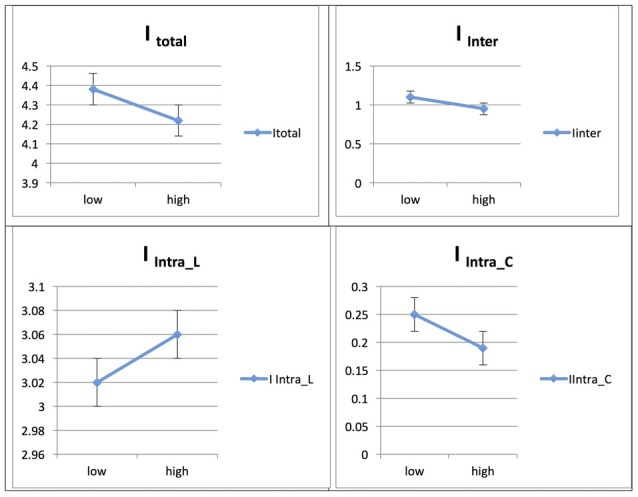
Probability value of the network integration differences across phases for L1 (Persian) in the Persian-speaking group. I = integration, I_Total_ = total integration value, I_Intra_Language_ = Intra-integration of language areas, I_Intra_Control_ = Intra-integration of cognitive control areas and I_Inter_ = integration between the two networks (language processing and cognitive control). A probability greater than 0.9 is considered significant.

See Figures 7, 8 for illustrations of network integration across phases for L1 and L2 in Persian speakers.

## Discussion

This study examined the influence of L2 word learning through verbal repetition on integration values within and between the language and cognitive control networks in a close and a distant language pair. Two groups of adults learned French (L2) through repetition and imitation by means of a computerized vocabulary-learning program. One group’s L1 was close to L2 (i.e., Spanish), whereas the other group’s L1 was distant from L2 (i.e., Persian). For each group, behavioral responses and functional integration values were collected at two points in time: after a week of training and after 4 weeks of training. Both groups received equivalent training of 15 min a day.

The results show a significant and equivalent improvement in behavioral responses (AR and RT for L2 naming) in both learning groups. Thus, both groups learned L2 words equally well in terms of accuracy and speed attained after training. With regard to network integration values, similarities and differences were observed across groups. Specifically, for Persian native speakers, integration values decreased between the language and control networks in L2, whereas these values remained stable for the L1 (Ghazi-Saidi et al., 2013). As for the Spanish speakers, there were no changes in integration values over time, neither with L1 nor with L2.

The behavioral results provide evidence of the efficacy of verbal repetition in L2 vocabulary learning, as shown by the significantly faster and more accurate performances obtained by both groups at the second learning phase. These observations are in line with previous work (e.g., Snedden, 1931; Bartels et al., 2010) on the efficacy of verbal repetition in novel vocabulary learning. Faster responses and better accuracy reflect increased automaticity following repetition (Forster and Chambers, 1973; Brown and Watson, 1987; Segalowitz and Hulstijn, 2005). Previous work shows that verbal repetition favors automaticity (Haier et al., 1992a,b; Raichle et al., 1994; Fischler, 1998; Segalowitz and Hulstijn, 2005), considered to be a sign of successful learning (Segalowitz and Frenkiel-Fishman, 2005). Thus, learning through repetition occurs by increased exposure to the stimulus, which favors automatic—thus faster and more accurate—processing (Logan, 1988). Furthermore, the results of this study show that—at least for vocabulary learning—the repetition strategy is equally efficient with close and distant language pairs. This result is consistent with past work on the efficacy of repetition in L2 learning, with both linguistically close and distant languages (e.g., Horst et al., 1998; Rott, 1999; Waring and Takaki, 2003; de la Fuente, 2006; Webb, 2007; Schmitt, 2008).

Efficacy of repetition in L2 vocabulary learning is reflected by functional connectivity patterns. Specifically in Persian native speakers, the decrease in functional integration values between the language and control networks at phase 2 shows that repetition decreases cognitive load (Abutalebi et al., 2005; Altarriba and Heredia, 2008; Leonard et al., 2011; Parker-Jones et al., 2011). The integration value reflects the information flow in the network. In other words, drilling through repetition induces neuroplastic changes reflected by the disengagement of the cognitive control network in sustaining L2 word retrieval in phase 2, in line with evidence from previous functional connectivity studies (Dodel et al., 2005; Prat et al., 2007; Majerus et al., 2008; Veroude et al., 2010).

Previous functional connectivity work by Dodel et al. (2005), Prat et al. (2007), Majerus et al. (2008) and Veroude et al. (2010), on different tasks in bilingual speakers report a decreased functional connectivity when processing demands increase. Specifically, functional connectivity decrease associated with increasing demands has been reported in reading tasks (Prat et al., 2007); syntactic processing load (Dodel et al., 2005); short-term memory demands (Majerus et al., 2008), and finally phonological processing (Veroude et al., 2010).

Previous studies in other cognitive domains, such as learning finger movement sequences, have shown that cognitive demand increases with task complexity (Robinson, 2001, 2011; Horberry et al., 2006; Coynel et al., 2009; Reimer, 2009; Linck et al., 2014), and that a reasonable cognitive load ensures engagement in the task, whereas an extremely high load can result in disengagement from the task (Wulf and Shea, 2002). In this study, the results with the Persian native group show that the complexity of the task was reasonable enough to require recruitment of the control system and keep it engaged throughout the learning process.

Another way of interpreting the results of this study invokes the notion of stimulus novelty, or how familiar the word is. Previous works (Petersson et al., 2000; Baddeley, 2003; Berthier et al., 2012; Berthier and Lambon Ralph, 2014) showed that verbal repetition may involve cognitive control abilities. Specifically, Berthier and Lambon Ralph (2014) argued that—in the context of aphasia therapy—neuroplastic changes related to the repetition of novel stimuli reflect cognitive control demands, an argument in line with previous studies on rehabilitation from traumatic brain injury (Kimberley et al., 2010), and motor learning experiments in healthy participants (Carey et al., 2005). Furthermore, the amount of cognitive control deployed in verbal repetition has been hypothesized to depend on word novelty (Chein and Schneider, 2005), as novel words consume more cognitive control resources than salient or familiar words. Converging evidence on the role of stimulus novelty comes from motor skill learning studies (Pascual-Leone et al., 1995; Sadato et al., 1996; Wulf and Shea, 2002; Hlustík et al., 2004; Carey et al., 2005; Kimberley et al., 2010) and language learning studies (Bialystok, 1999; Robinson, 2005; Ghazi-Saidi et al., 2013; Calvo and Bialystok, 2014; Ghazi-Saidi and Ansaldo, 2016).

In the present study, the Persian group showed changes in integration values between the language and control networks over time and with L2 only, whereas in the Spanish native group, no changes over time were observed in either language. We argue that the degree of overlap between L1 and L2 items—which is a function of L1-L2 distance—determines how novel L2 items are. More specifically, although both groups were exposed to an equal amount of repetition and achieved equal behavioral performance, in the group with a close pair of languages, stimuli were more salient—or less novel—and thus did not require the recruitment of the cognitive control system. Conversely, in the Persian native group, the novelty of L2 items was higher due to the lesser degree of L1-L2 overlap, and thus the load on the cognitive control system was higher. Hence, these results show that the absence of behavioral effects of repetition does not preclude differences at the network level. In the present case, repetition resulted in equivalent performance across experimental groups, but the persistent engagement of the cognitive control network in Persian speakers reflects effortful processing due to language distance effects. Hence, these results show that the impact of repetition on functional connectivity patterns is modulated by changes at the network level, even in the absence of behavioral differences across the experimental groups. These results have implications for L2 teaching and learning, as well as for interventions in cases of bilingual aphasia. Language learners, teachers and therapists should therefore consider the impact of language distance when setting a minimum number of repetitions for learning new words. In the Communicative Language Teaching approach, practice in the form of drilling and repetition is usually banned, as this is considered mechanical and unnatural. The present study brings neuroimaging evidence that in the case of distant L2 words drilling can be beneficial to improve proficiency. However, with distant languages, the amount of repetition or the amount of practice should increase to achieve optimal performance.

## Conclusion

The results of the present study provide neurofunctional evidence of the effects of repetition as a neuroplasticity agent in L2 learning. While L2 integration values between language and control networks decreased over time in the group with a distant language pair (Persian and French), no changes in integration values within or across languages or networks were observed in the group with a close language pair (Spanish and French). Novelty is higher for L2 words of distant language pairs than in close language pairs (Ringbom, 2007); hence, processing a close L2 vocabulary consumes less cognitive control resources than processing a distant L2, from the very beginning of the learning process; conversely, cognitive demands with a distant L2 remain strong, even when the L2 vocabulary has been consolidated.

These results show that repetition effects in L2 learning are modulated by word novelty, a factor that depends upon L1-L2 distance. Implications for L2 teaching and intervention plans with bilingual patients concern the amount of repetition required to reduce cognitive load as a function of L1-L2 distance. Further studies of the modulatory effect of language distance on repetition effects on L2 learning are required.

## Author Contributions

LG-S: Recruitment and data acquisition, data analysis, interpretation, drafting, revision and edition of the manuscript. AIA: supervision, revision and edition of the manuscript.

## Conflict of Interest Statement

The authors declare that the research was conducted in the absence of any commercial or financial relationships that could be construed as a potential conflict of interest.

## Footnotes

^1^http://www.neurobs.com

^2^http://www.fil.ion.ucl.ac.uk/spm

^3^http://sites.google.com/site/netbrainwork/
